# Delay of Early B-Lymphocyte Development by Gamma 2b Immunoglobulin Transgene: Effect on Differentiation-Specific Molecules

**DOI:** 10.1155/1990/71584

**Published:** 1990

**Authors:** Kathleen A. Denis, Scott Provost, Owen N. Witte, Ralph L. Brinster, Ursula Storb

**Affiliations:** 1 Howard Hughes Medical Institute UCLA Los Angeles California 90024 USA; 2 School of Veterinary Medicine University of Pennsylvania Philadelphia Pennsylvania 191104 USA; 3 Department of Molecular Genetics and Cell Biology University of Chicago Chicago Illinois 60637 USA

## Abstract

Mice transgenic for γ2b Ig heavy chain were examined for alterations in B-cell differentiation and endogenous Ig gene rearrangement and expression. Fresh bone marrow from these mice was markedly reduced in BP-1^+^ cells and there were small reductions in B220^+^ and sIg^+^ cells. A-MuLV (Abelson murine leukemia virus) transformants from these bone marrow cells showed little alteration in Ig gene rearrangement and expression when compared to controls, however. Isolation of the B-lymphoid compartment from these mice in vitro using LBMC (lymphoid bone marrow cultures) enabled more detailed characterization of the effects of the transgene. LBMC derived from γ2b transgenic mice had similar growth kinetics, but a 4-5-week delay in the expression of endogenous mu Ig in comparison to control cultures. Nucleic acids derived from these early cultures prior to endogenous mu Ig expression showed reduced Ig J_H_ rearrangements, some sterile mu transcription, low levels of BP-1 expression, and virtually undetectable TdT (terminal deoxynucleotidyl transferase) expression. Thus, this γ2b transgene appears able to affect early B-lymphocyte development.


*Developmental Immunology*, 1990, Vol. 1, pp. 105–112
Reprints available directly from the publisher
Photocopying permitted by license only

© 1990 Harwood Academic Publishers GmbH
Printed in the United Kingdom

### Delay of Early B-Lymphocyte Development by Gamma 2b Immunoglobulin Transgene: Effect on Differentiation-Specific Molecules

KATHLEEN A. DENIS[*,+], SCOTT PROVOST[‡], OWEN N. WITTE[+], RALPH L. BRINSTER[§], and URSULA STORB[‡]

[+]*Howard Hughes Medical Institute, UCLA, Los Angeles, California 90024*
[§]*School of Veterinary Medicine, University of Pennsylvania, Philadelphia, Pennsylvania 191104*
[‡]*Department of Molecular Genetics and Cell Biology, University of Chicago, Chicago, Illinois 60637*

Mice transgenic for γ2b Ig heavy chain were examined for alterations in B-cell differentiation and endogenous Ig gene rearrangement and expression. Fresh bone marrow from these mice was markedly reduced in BP-1$^+$ cells and there were small reductions in B220$^+$ and sIg$^+$ cells. A-MuLV (Abelson murine leukemia virus) transformants from these bone marrow cells showed little alteration in Ig gene rearrangement and expression when compared to controls, however. Isolation of the B-lymphoid compartment from these mice in vitro using LBMC (lymphoid bone marrow cultures) enabled more detailed characterization of the effects of the transgene. LBMC derived from γ2b transgenic mice had similar growth kinetics, but a 4–5-week delay in the expression of endogenous mu Ig in comparison to control cultures. Nucleic acids derived from these early cultures prior to endogenous mu Ig expression showed reduced Ig $J_H$ rearrangements, some sterile mu transcription, low levels of BP-1 expression, and virtually undetectable TdT (terminal deoxynucleotidyl transferase) expression. Thus, this γ2b transgene appears able to affect early B-lymphocyte development.

KEYWORDS: Immunoglobulin genes, transgene, B lymphocyte development, long-term lymphoid cultures.

### INTRODUCTION

Mice transgenic for immunoglobulin genes have been utilized to study the control of expression of these genes as well as the regulation of the encoded antibody molecules (reviewed in Storb, 1988). In addition to specific effects upon endogenous Ig genes, transgenes can exert more global influences on early B-lymphocyte development. Two different lines of mice transgenic for mu heavy-chain genes have been shown to lack the major population of bone marrow derived B cells (Herzenberg et al., 1987) or have an altered pre-B-cell compartment (Nussenzweig et al., 1988), indicating a perturbation in early B-cell development.

We have investigated the nature of the changes in the early B-lymphocyte lineage cells in Ig γ2B heavy-chain transgenic mice (Tsang et al., 1988) by evaluating this developmental compartment using lymphoid bone marrow cultures (LBMC, Whitlock and Witte, 1982; reviewed in Denis and Witte, 1989). These cultures allow the study of B-lymphocyte commitment and differentiation from a multipotential progenitor in vitro (Denis and Witte, 1986; Dorshkind, 1986; Muller-Sieburg et al., 1986). Mice transgenic for a functionally rearranged γ2b Ig heavy chain were utilized for these studies. Cells expressing endogenous mu could be easily discriminated from those expressing the transgene, obviating the need for allotypic analysis (Herzenberg et al., 1987) or interspecies studies (Nussenzweig et al., 1988).

These γ2b transgenic mice were deficient in bone marrow pre-B cells, much as has been shown with other transgenic lines. LBMC established from these mice demonstrated a delayed expression of endogenous Ig as well as delayed rearrangement at the IgH locus when compared to control cultures. Despite the presence of transcription at the IgH locus,

*Corresponding author. Present address: Specialty Laboratories, 2211 Michigan Avenue, Santa Monica, California 90404.

terminal deoxynucleotidyl transferase (Alt and Baltimore, 1982; Landau et al., 1987) expression was markedly reduced. The transcription of another early B-lymphocyte-development molecule, BP-1 (Cooper et al., 1986), was also reduced in these cultures. The presence of this $\gamma 2b$ transgene may affect B-lymphocyte differentiation as well as Ig recombination.

### RESULTS

### The Bone Marrow of $\gamma 2b$ Transgenic Mice Has a Significantly Reduced Pre-B-Cell Compartment

Bone marrow cells of $\gamma 2b$ transgenic mice were examined by flow cytometry to define their lymphoid composition in comparison to that of bone marrow from normal littermates. Fresh femoral bone marrow was harvested from these mice and stained with FITC-labeled reagents for sIgM, sIgG2b, B220 (Kincade, 1981) and BP-1 (Cooper et al., 1986). These markers were chosen to give an overview of the B-cell-differentiation stages present. B220 is expressed by almost all B-lymphoid cells (Kincade et al., 1989), whereas BP-1 (equivalent to 6C3; Wu et al., 1989) is present only on late B precursors and pre-B cells in the bone marrow. The more mature B-cell population expresses sIgM while sIgG2b expression is rare in normal bone marrow, but present in a subpopulation of B cells transgenic for the $\gamma 2b$ heavy chain. As summarized in Table 1, the bone marrow cells from the $\gamma 2b$ transgenic mice had decreased expression of the B-lineage markers, BP-1, B220, and sIgM. The sIgM+ and B220+ cells are two-fold reduced in the bone marrow of these transgenic mice, but the largest perturbation is in the BP-1$^+$ population. These early B-lymphoid cells are reduced five- to ten-fold in the $\gamma 2b$ transgenic mouse bone marrow.

TABLE 1
Cell-Surface Staining of Bone Marrow from $\gamma 2b$ Transgenic Mice and Normal Litermates[a]

| | Normal | Transgenic |
|---|---|---|
| BP-1 | 13.5% (12–15) | 2.0% (1–3) |
| B220 | 20.3% (19–22) | 14.3% (12–16) |
| IgM | 12.5% (9–15) | 4.3% (2–6) |
| IgG2b | 1.2% (0.5–2) | 5.0% (4–6) |

[a]Data are presented as the mean (range) of three independent experiments. Fresh bone marrow from mice 5 to 10 weeks of age was stained directly with FITC-labeled antimouse IgM or antimouse IgG2b (Southern Biotech, Birmingham, AL) or indirectly with BP-1 (generously provided by Dr. M. D. Cooper) or B220 (14.8) followed by FITC-labeled antimouse IgG2a or antirat Ig, respectively. Cells were analyzed on a Coulter Epics.

To further analyze the early B-lymphoid compartment of these mice, cell lines were derived by a A-MuLV transformation and single-cell cloning of bone marrow. As seen in Table 2, only a slight reduction of A-MuLV target cells was observed in the $\gamma 2b$ transgenic mouse bone marrow. This is not surprising, as the vast majority of A-MuLV target cells in normal bone marrow are BP-1 (6C3) negative but B220$^+$ (Tidmarsh et al., 1989). By using metabolic labeling followed by immunoprecipitation and SDS-polyacrylamide gel electrophoresis to identify the Ig chains synthesized by each cell line, it could be shown that all of the cell lines derived from transgenic mice were synthesizing gamma heavy chain (Table 2). In addition, 2 of 12 of these lines coexpressed mu and one of these also expressed kappa. The cell lines derived from the bone marrow of normal litter mates correlated well with the range of Ig expression previously observed in A-MuLV-transformed cells from normal bone marrow (Alt et al., 1981) with 40% mu+, 10% kappa+ and no gamma+ lines (Table 2).

TABLE 2
A-MuLV-Transformed Cell Lines Derived from the Bone Marrow of $\gamma 2b$ Transgenic Mice and Normal Littermates[a]

| | Normal | Transgenic |
|---|---|---|
| A-MuLV target frequency (per $10^6$ cells) | 12.1 | 8.7 |
| Ig synthesized | | |
| gamma | 0% | 100% |
| mu | 40% | 17% |
| kappa | 10% | 8% |
| IgH Gene Configuration | | |
| E | 0% | 0% |
| DJ | 15% | 33% |
| VDJ | 85% | 58% |

[a]Fresh bone marrow was transformed with A-MuLV and cloned in agar as previously described (Whitlock et al., 1983). Data are derived from the analysis of 12 transgenic and 10 normal littermate-derived cell lines, as described in the text. The IgH gene configuration is the percent of total alleles; 2/24 alleles in the transgenic cell lines were deleted. A 5′ D probe was used to differentiate DJ from VDJ rearrangements (Alt et al., 1984).

The IgH gene configuration of these cell lines was also examined using a $J_H$ probe (Early et al., 1980), a 5′ of $J_H$ probe (Weaver et al., 1985) and a 5′D probe (Alt et al., 1984). It has been demonstrated that the initial event that takes place in the heavy-chain locus is a D segment to J region rearrangement event, and the presence of the 5′ $J_H$ region or the 5′D region indicates that D–J or V–D rearrangement, respectively, has not yet taken place (Alt et al., 1984). By using these analyses, it was shown that all cell lines had undergone at least D–J rearrangement (Table 2). The transgenic cell lines had approximately twice as many D–J only rearrangements as did the normal control. These results were in contrast to the finding of complete inhibition of endogenous D–J rearrangement seen in some pre-B cells or in hybridomas of transgenic mice with membrane terminus containing mu (Rusconi and Kohler, 1985; Weaver et al., 1985; Nussenzweig et al., 1987; Manz et al., 1988) or delta (Iglesias et al., 1987) transgenes. However, over 30% of hybridomas from spleen B cells of the same γ2b transgenic mice show one germline H allele (Roth and U.S., unpublished). Thus, γ2b can exert feedback inhibition of Ig gene rearrangement (see what follows).

### LBMC from γ2b Transgenic Mice Have Delayed Expression of Endogenous Ig

The slight perturbation of endogenous Ig gene rearrangement and expression seen in the A-MuLV transformants from the γ2b transgenic mice did not appear to reflect the changes observed in the fresh bone marrow. We therefore turned to LBMC to isolate a wider spectrum of early B-lymphoid development from the bone marrow of these mice. Long-term lymphoid cultures (Whitlock and Witte, 1982; Whitlock et al., 1984) derived from the bone marrow of γ2b transgenic and normal littermates appeared kinetically and morphologically similar in the development of their adherent and nonadherent cell compartments. After several weeks of culture, both the transgenic and normal LBMC had confluent lawns of nonadherent lymphoid cells of approximately the same cell density that were sampled and examined for Ig synthesis by immunoperoxidase cell staining. The lymphoid cells from the cultures established from the bone marrow of the normal littermates were an average of 13% positive for mu heavy chain after 4 weeks of culture (Fig. 1) and rose to 20–30% positive by 5–6 weeks of culture. These values are very typical for LBMC established from BALB/c bone marrow (Denis et al., 1984). No expression of gamma heavy chain was seen in these cultures. In contrast, the lymphoid cells from cultures established from the bone marrow of the γ2b transgenic mice were less than 1% positive for mu heavy chain after 4 weeks of culture and did not

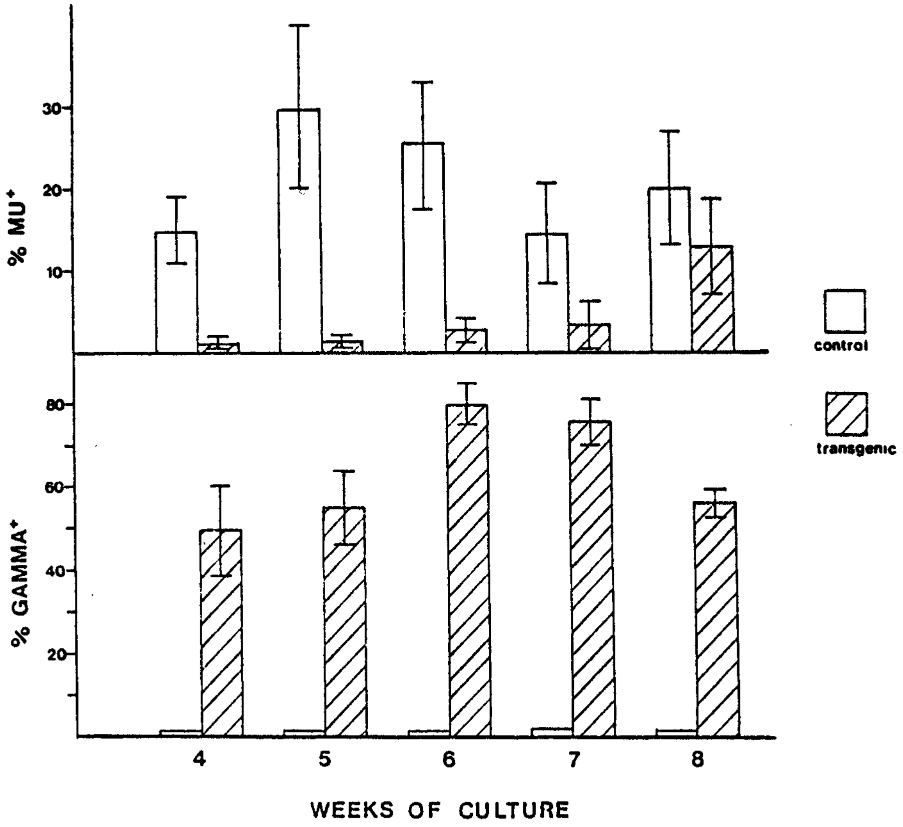

FIGURE 1. Cytoplasmic Ig production in cells from LBMC. Cytospin preparations of cells from LBMC derived from either γ2b transgenic or normal control mouse bone marrow were made at weekly intervals. These were stained with Ig-specific reagents, as described in the text. The results are the mean and standard deviation of 6–12 culture dishes derived from 2-5 individual mice in each group.

reach a level of more than 10% positive until 8 weeks of culture. During this time, the transgenic γ2b heavy chains were expressed by a majority of the cells in the LBMC.

After 5 weeks of culture, when mu heavy-chain production was very low in LBMC derived from γ2b transgenic mice, cells were harvested and DNA extracted. This DNA was analyzed by Southern blot for the presence of rearrangements at the $J_H$ locus. Numerous rearrangements of the $J_H$ locus could be seen in the DNA from the normal LBMC with loss of intensity of the 6.2-kb germ-line fragment (Fig. 2). As a population, the LBMC derived from the γ2b transgenic mice showed a much greater proportion of germ-line $J_H$ region fragment (Fig. 2, lane C). A mu probe was used to demonstrate equivalent DNA loading (data not shown). This would indicate that the delay in mu Ig heavy-chain production is the result of decreased rearrangement activity in the transgenic bone marrow derived LBMC.

Following 8–10 weeks of culture, the levels of endogenous Ig expressed by the normal and transgenic LBMC were approximately equal. To assess the size and charge diversity of these Ig chains, two-dimensional gel electrophoresis was performed. The nonadherent cells were harvested and labeled with $^{35}$S-methionine. Gels were run following lysis and immunoprecipitation of the radio-labeled Ig. As shown in Fig. 3, both types of LBMC were producing multiple species of mu and light chains. The transgenic LBMC were also synthesizing a monoclonal gamma chain corresponding to that encoded by the transgene. It appeared that the presence of the transgene did not restrict the synthesis of a variety of mu and light chains, although there seemed to be somewhat fewer species than synthesized by the control cultures. This may indicate pauciclonality due to selection of a subpopulation of mu-producing pre-B cells.

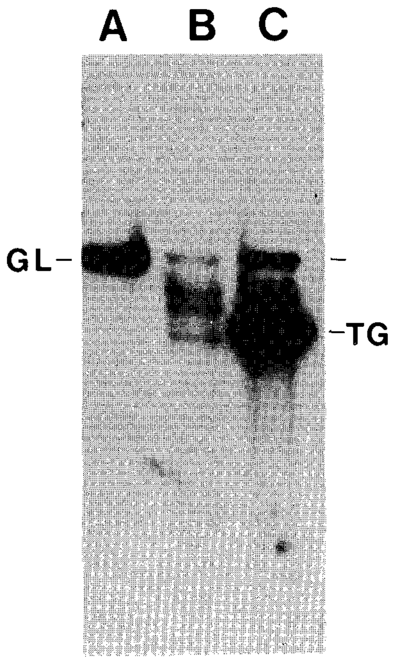

FIGURE 2. $J_H$ rearrangements of DNA from LBMC by Southern blot. DNA (10 μg) from mouse liver (A), control LBMC (B), or γ2b transgenic LBMC after 5 weeks of culture (C) were digested with EcoRI. Hybridization is with a $J_H$ probe; the unrearranged $J_H$ germ-line band (GL) is shown on either side of the autoradiogram. The probe also hybridizes to the integrated transgene (TG).

To demonstrate that single cells from the transgenic LBMC were synthesizing mu, gamma, and kappa chains, clonal lines were derived with

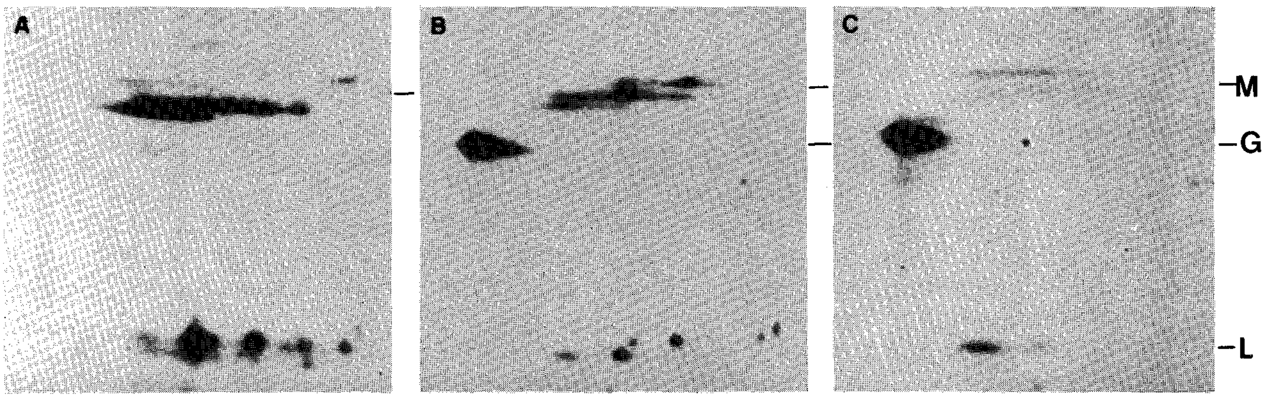

FIGURE 3. Two-dimensional gel electrophoresis of cell lysates. Equal numbers of cells from control (panel A) or γ2b transgenic (panel B) mouse-derived LBMC or an A-MuLV-derived clonal line from γ2b transgenic LBMC (panel C) were labeled with $^{35}$S-methionine in the presence of tunicamycin. Ig chains were analyzed in the first dimension by a pH 8-4 isoelectric focusing gel (left to right) and in the second dimension on a NaDod $SO_4$/10% polyacrylamide gel. Mu (M), gamma (G), and light chain (L) are shown.

A-MuLV transformation and agar cloning. Coproduction of mu, gamma, and kappa were seen in five of six lines, as evaluated by two-dimensional gel analysis (Fig. 3, panel C). The sixth cell line did not produce gamma heavy chain, and DNA analysis showed deletion of the transgene (data not shown). Thus, although the presence of the transgenic $\gamma$2b heavy chain appeared to delay rearrangement and expression of endogenous Ig heavy chains, eventually cells were able to express heterogeneous mu and kappa chains and often coexpressed these with the transgenic $\gamma$2b chains.

### LBMC from $\gamma$2b Transgenic Mice Have Sterile Mu Transcription without TdT Expression

To address the mechanism of the delay in IgH gene rearrangement and expression, two known intermediate steps in this process were evaluated. First, the production of germ-line Ig transcripts in the early LBMC cells was monitored. The initial step of the Ig gene recombination process is the transcriptional activation of these genes (Yancopoulos and Alt, 1986; Schuler et al., 1988). This presumably reflects the accessibility of the Ig genes to the recombinase enzymes. The presence of germ-line transcripts would indicate the commitment of these LBMC cells to the B-lymphocyte lineage.

Cytoplasmic mRNA was isolated from 5–6-week-old LBMC derived from normal and $\gamma$2b transgenic bone marrow. This mRNA was analyzed by Northern blotting following agarose-formaldehyde gel electrophoresis. Hybridization with a mu Ig heavy-chain consant region probe showed that the transgenic LBMC cells contained transcripts of 3.0 and 1.9 kb, the size of sterile mu transcripts produced from unrearranged mu genes (Fig. 4, lane D in the first panel) (Alt et al., 1982; Schuler et al., 1988). These could be seen in the control cell line in lane B, which is an A-MuLV transformant of bone marrow from a severe combined immunodeficient mouse that has two germ-line IgH (Denis unpublished). LBMC cells from the normal cultures also contained sterile mu transcripts, but additionally produced 2.7- and 2.4-kb transcripts indicative of intact, rearranged mu heavy-chain mRNA (Figure 4, lane C in the first panel). These data would suggest that the Ig genes in both of the LBMC-derived cells were transcriptionally active, but only the normal-littermate-derived cultures had recombined to form functional mu heavy-chain genes at that time.

Second, these same mRNAs were evaluated for the presence of TdT, a molecularly cloned enzyme of the recombinase process (Desiderio et al., 1984; Landau et al., 1987). Following transcriptional activation of the IgH genes, recombinase enzymes would be activated to effect recombination. Hybridization of the Northern blot with a TdT probe (Landau et al., 1987) detected no TdT transcripts in the LBMC cells derived from $\gamma$2b transgenic mice (Figure 4, panel 2). Control LBMC were highly posi-

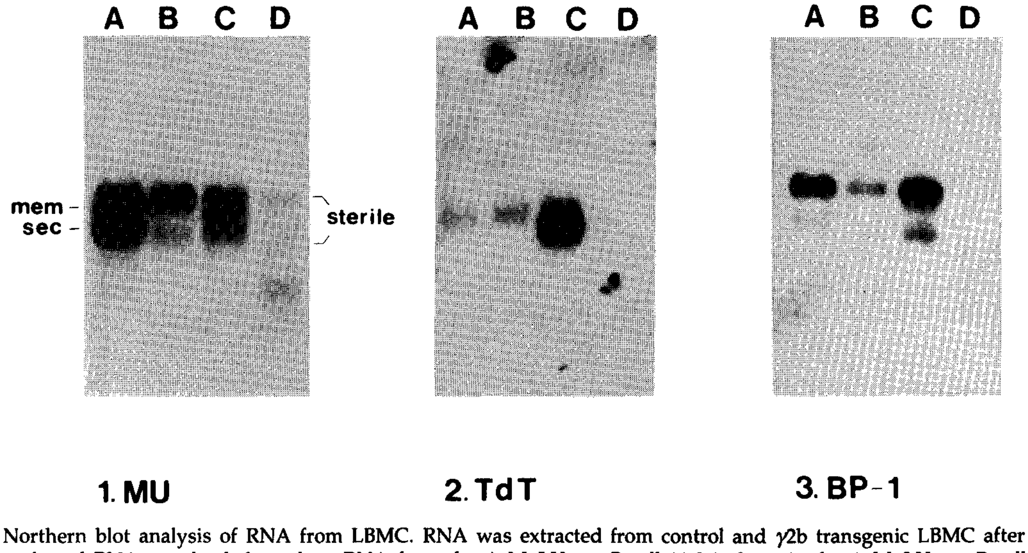

FIGURE 4. Northern blot analysis of RNA from LBMC. RNA was extracted from control and $\gamma$2b transgenic LBMC after 5 weeks of culture. 20 $\mu$g of total RNA was loaded per lane RNA from the A-MuLV pre-B cell 18.8 in lane A, the A-MuLV pre-B cell from SCID bone marrow in lane B, control LBMC in lane C, and $\gamma$2b transgenic LBMC in lane D. Blot 1 is probed with $\gamma$12 (Rogers et al., 1980), blot 2 with TdT (Landau et al., 1987), and blot 3 with a probe for BP-1 (kindly provided by M. D. Cooper).

tive as were early lymphoid-control cell lines tested. The Northern blot was also probed for glyceraldehyde 3-phosphate dehydrogenase gene expression (GAPDH; Piechazyk et al., 1984), used as a measure of mRNA quantity and integrity. This demonstrated equal amounts of mRNA in the control and transgenic LBMC-derived lanes (data not shown).

To determine if TdT in early T lymphocytes was also decreased, mRNA was extracted from the thymuses of young γ2b transgenic and normal littermate mice. High TdT levels were observed in both types of mice by Northern blot analysis (data not shown). The γ2b transgene is transcribed in thymocytes (Tsang et al., 1988), but does not appear to affect TdT expression in these cells by this analysis.

Finally, the expression of BP-1, a specific marker of early B lymphocytes important in their differentiation (Cooper et al., 1986) was evaluated. As shown in Fig. 4, panel 3, the expression of BP-1 in the LBMC derived from the bone marrow of γ2b transgenic mice was also markedly reduced compared to control LBMC. Thus, the γ2b gene appeared to exert a more global effect on B-lymphocyte differentiation.

### DISCUSSION

The processes of B-lymphocyte differentiation and Ig gene expression are firmly intertwined. In this paper, we have demonstrated that mice transgenic for the γ2b Ig heavy chain have an altered early B-cell compartment in their bone marrow. A strong effect on peripheral B cells is also seen in young γ2b transgenic mice. However, adult mice with the 343-1 γ2b transgene have a relatively normal peripheral B-cell phenotype (P. Roth and U.S., unpublished). Even A-MuLV transformants of the bone marrow of these mice were only slightly altered in their Ig expression and gene rearrangement status (Table 2).

Isolation of the B-lymphocyte developmental compartment of these mice using long-term lymphoid cultures enabled us to view events that are rare or short-lived in vivo and expand them for molecular analysis. LBMC from the transgenic mice had delayed expression of endogenous mu heavy chain. Despite being transcriptionally active, the mu heavy-chain locus was unable to rearrange efficiently in these cultured cells during the first few weeks. This may be the result of feedback inhibition on the recombinase pathway enzymes exemplified by a paucity of TdT expression. Indeed, preliminary results by Northern blot analysis indicate that these early transgenic LBMC cells have decreased expression compared to controls of a second recombinase associated enzyme RAG-1 (Schatz et al., 1989; Schatz and Baltimore, personal communication).

At this early culture time point, the expression of BP-1 molecules was also reduced. This may suggest that the presence of an Ig transgene can affect differentiation molecules other than endogenous Ig and the enzymes utilized in the Ig recombination and expression pathway. This decrease in BP-1-expressing cells was also observed in fresh bone marrow from the γ2b transgenic animals. After several additional weeks of culture, the transgenic LBMC were populated by cells that had successfully rearranged and were expressing their mu heavy-chain genes.

This delay in Ig expression could be due to slowed differentiation of normal numbers of precursors or differentiation from decreased numbers of precursors due to an inhibitory mechanism such as toxicity of the γ2b transgene in early cells. Despite similar growth kinetics in the normal and transgenic LBMC, these cultures lack certain negative regulatory elements and cells are allowed to survive that might be deleted in vivo. A parallel can be drawn with LBMC derived from severe combined immunodeficient mice (SCID; Witte et al., 1987). The nonadherent cells that developed in these cultures were identical to control cultured cells in the expression of a variety of surface markers. However, the SCID-derived cells did not express Ig and had numerous aberrant Ig gene rearrangements. These cells, which must be selectively eliminated in the intact animal, were allowed to proliferate in the LBMC environment. In the γ2b transgenic mice, the B lymphocytes with delayed Ig recombination are perhaps eliminated in such a manner, making them difficult to isolate from the intact animal and resulting in a reduced pre-B-lymphocyte compartment. However, in the LBMC, because of the absence of negative regulation, the cells are permitted to live. The cells that reach the periphery in the adult γ2b transgenic mice have either overcome this block in Ig gene expression or arise from a different cell lineage. Several γ2b transgenic mouse lines appear to have the same phenotype as the 343-1 line described here (Lo, Roth, Doglio, and U.S., unpublished). However, another line of γ2b transgenic mice has a much different phenotype, with reduced mu+B-cell numbers in the periphery (Lo, Roth, Doglio, and

U.S., unpublished). It will be of great interest to examine the peripheral lymphoid compartment of these animals and observed B-lymphocyte development in the neonate and young adult.

### MATERIALS AND METHODS

### Transgenic Mice

The $\gamma$2b transgenic mice have been described previously (Tsang et al., 1988). In this study only, the 343-1-13 line was utilized.

### Fluorescence Analysis

Fresh bone marrow cells were harvested from $\gamma$2b transgenic and normal littermate mice and stained with FITC-labeled reagents as described in Dorshkind et al. (1986). BP-1 (a kind gift from M. D. Cooper) staining was indirectly detected with antimouse $\gamma$2a (Southern Biotech, Birmingham, AL); B220 staining was detected with antirat Ig (Southern Biotech). IgM and IgG2b surface staining was performed directly. Cells were analyzed on an Epics V flow cytometer (Coulter, Hialeah, FL).

### Abelson Murine Leukemia Virus (A-MuLV) Tranformants

Bone marrow cells from gamma 2b transgenic mice and their normal littermates (Tsang et al., 1988) were taken at 3 weeks of age and infected with A-MuLV (Rosenberg and Baltimore, 1976). Briefly, $3 \times 10^6$ cells were infected with A-MuLV p120 viral stock (Witte, 1983) in the presence of 0.8 $\mu$l/ml of polybrene (Sigma, St. Louis, MO) at 37°C for 3 h. The cell suspension was then washed and plated in soft agar as previously described (Whitlock et al., 1983). Clonal outgrowths were visible 10–14 days later and were plucked from agar using a pasteur pipet and expanded for analysis.

### Lymphoid Bone Marrow Cultures

Bone marrow cells from the transgenic and control animals were taken at 3 weeks of age and plated at $1 \times 10^6$ cells per ml in 5 ml total volume in 6-cm tissue culture plates (#25010, Corning Glass Works, Corning, NY). These cultures were then maintained as previously described (Whitlock et al., 1984).

### Metabolic Labeling, Immunoprecipitation, and Gel Electrophoresis

Metabolic labeling of the cultured cells with $^{35}$S-methionine (Amersham, Arlington Heights, IL) in the presence of tunicamycin (Sigma),immunoprecipitation with goat antimouse Ig (Boehringer Mannheim Biochemicals, Indianapolis, IN), and SDS-polyacrylamide or two-dimensional electrophoresis were performed, as previously described (Denis et al., 1984).

### Nucleic Acid Extraction and Analysis

Nucleic acids were extracted from tissues and cell lines according to established procedures (Maniatis et al., 1982; Davis et al., 1986). Southern blot analysis was performed, as previously described (Denis et al., 1984). Northern blot analysis was done as previously described (Tsang et al., 1988). Nucleic acid probes used for these analyses are described in the appropriate figure legends.

### Immunoperoxidase Cell Staining

Cytocentrifuge preparations of cells were stained with peroxidase-conjugated goat antimouse mu heavy chain (Boehringer-Mannheim) or gamma heavy chain (Kierkegaard and Perry Laboratories, Gaithersburg, MD), as previously described (Dēnis et al., 1984).

### ACKNOWLEDGMENTS

The authors would like to thank Drs. M. D. Cooper, D. G. Schatz, and D. Baltimore for sharing reagents and their unpublished results, and Dr. D. Lo for helpful discussions. We are grateful for the expert technical assistance of E. Spoor and L. Timson and for the word processing skills of C. Crookshank.

This work was supported by the Howard Hughes Medical Institute and by grant HD23089 from the National Institutes of Health.

*(Received February 16, 1990)*

*(Accepted May 5, 1990)*

### REFERENCES

Alt F.W., and Baltimore D. (1982). Joining of immunoglobulin heavy chain gene segments: Implications from a chromosome with evidence of three D-$J_H$ fusions. Proc. Natl. Acad. Sci. USA **79:** 4118–4122.

Alt F.W., Rosenberg N., Enea V., Siden E., and Baltimore D. (1982). Multiple immunoglobulin heavy chain gene transcripts in Abelson murine leukemia virus transformed lymphoid cell lines. Mol. Cell. Biol. **2:** 386–400.

Alt F., Rosenberg N., Lewis S., Thomas E., and Baltimore D. (1981). Organization and reorganization of immunoglobulin genes in A-MuLV transformed cells: Rearrangement of heavy but not light chain genes. Cell **27:** 381–390.

Alt F.W., Yancopoulos G.D., Blackwell T.K., Wood C., Thomas E., Boss M., Coffman R., Rosenberg N., Tonegawa S., and Baltimore D. (1984). Ordered rearrangement of immunoglobulin heavy chain variable region segments. EMBO J. **3:** 1209–1219.

Cooper M.D., Mulvaney D., Coutinho A., and Cazenave P.A. (1986). A novel cell surface molecule on early B-lineage cells. Nature **321:** 616–618.

Davis L.G., Dibner M.D., and Baltey J.F. (1986). Basic Methods in Molecular Biology (New York: Elsevier Science Publishing Co., Inc.).

Denis K.A., Treiman L.J., St. Claire J.I., and Witte O.N. (1984). Long term cultures of murine fetal liver retain very early B lymphoid phenotype. J. Exp. Med. **160:** 1087–1101.

Denis K.A., and Witte O.N. (1986). *In vitro* development of B lymphocytes from long-term cultured precursor cells. Proc. Natl. Acad. Sci. USA **83:** 441–445.

Denis K.A., and Witte O.N. (1989). Long term lymphoid cultures in the study of B-cell differentiation. *In:* Immunoglobulin Genes, Hanjo T., Alt F.W., and Rabbitts T.H., Eds. (New York: Academic Press), pp. 45–59.

Desiderio S.V., Yancopoulos G.D., Paskind M., Thomas E., Boss M.A., Landau N., Alt F.W., and Baltimore D. (1984). Insertion of N regions into heavy-chain genes is correlated with expression of terminal deoxytransferase in B cells. Nature **311:** 752–755.

Dorshkind, K. (1986). *In vitro* differentiation of B lymphocytes from primitive hemopoietic precursors present in long-term bone marrow cultures. J. Immunol. **136:** 422–429.

Dorshkind K., Denis K.A., and Witte, O.N. (1986). Lymphoid bone marrow cultures can reconstitute heterogenous B and T cell dependent responses in severe combined immunodeficient mice. J. Immunol. **137:** 3457–3463.

Early P., Huang H., Davis M., Calame K., and Hood L. (1980). An immunoglobulin heavy chain variable region gene is generated from three segments of DNA: $V_H$, D and $J_H$. Cell **19:** 981–992.

Herzenberg L.A., Stall A.M., Braun J., Weaver D., Baltimore D., and Grosschedl R. (1987). Depletion of the predominant B-cell population in immunoglobulin $\mu$ heavy-chain transgenic mice. Nature **329:** 72–74.

Iglesias A., Lamers M., and Kohler G. (1987). Expression of immunoglobulin delta chain causes allelic exclusion in transgenic mice. Nature **330:** 482–484.

Kincade P.W. (1981). Formation of B-lymphocytes in fetal and adult life. Adv. Immunol. **31:** 177–245.

Kincade P.W., Lee G., Pietrangeli C.E., Hayashi S.-I., and Gimble J.M. (1989). Cells and molecules that regulate B lymphopoiesis in bone marrow. Annu. Rev. Immunol. **7:** 111–143.

Landau N.R., Schatz D.G., Rosa M., and Baltimore D. (1987). Increased frequency of N-regional insertion in a murine pre-B cell line infected with a terminal deoxynucleotidyl transferase retroviral expression vector. Mol. Cell. Biol. **7:** 3237–3243.

Maniatis T., Fritsch E.F., and Sambrook J. (1982). Molecular Cloning—A Laboratory Manual. (Cold Spring Harbor, NY: Cold Spring Harbor Laboratory).

Manz J., Denis K., Witte O., Brinster R., and Storb U. (1988). Feedback inhibition of immunoglobulin gene rearrangement by membrane $\mu$, but not by secreted $\mu$ heavy chains. J. Exp. Med. **168:** 1363–1381.

Muller-Sieburg C.E., Whitlock C.A., and Weissman I.L. (1986). Isolation of two early B lymphocyte progenitors from mouse bone marrow: A committed pre-pre-B cell and a clonogenic Thy-1$^{lo}$ henatopoietic stem cell. Cell **44:** 653–662.

Nussenzweig M.D., Schmidt E.V., Shaw A.C., Sinn E., Campos-Torres J., Mathey-Prevot B., Pattengale P.K., and Leder P. (1988). A human immunoglobulin gene reduces the incidence of lymphomas in c-*myc* bearing transgenic mice. Nature **336:** 446–450.

Nussenzweig M.C., Shaw A.C., Sinn E., Danner D.B., Holms K.L., Morse H.C., III, and Leder P. (1987). Allelic exclusion in transgenic mice that express the membrane form of immunoglobulin $\mu$. Science **236:** 816–819.

Piechazyk M., Blanchard J.M., Marty L., Dani C., Panabieres F., Sabouty S., Fort P., and Jeanteur P. (1984). Post-transcriptional regulation of glyceraldehyde-3-phosphate-dehydrogenase gene expression in rate tissues. Nucleic Acids Res. **12:** 6951–6963.

Rogers J., Early P., Carter C., Calame K., Bond M., Hood L., and Wall R. (1980). Two mRNAs with different 3′ ends encode membrane bound and secreted forms of immunoglobulin $\mu$ chain. Cell **20:** 303–312.

Rosenberg N., and Baltimore D. (1976). A quantitative assay for the transformation of bone marrow cells by Abelson murine leukemia virus. J. Exp. Med. **143:** 1453–1463.

Rusconi S., and Kohler G. (1985). Transmission and expression of a specific pair of rearranged immunoglobulin $\mu$ and kappa genes in a transgenic mouse line. Nature **314:** 330–334.

Schatz D.G., Oettinger M.A., and Baltimore D. (1989). The V(D)J recombination activating gene, RAG-1. Cell **9:** 1035–1048.

Schuler W., Schuler A., Lennon G.G., Bosma G.C., and Bosma M.J. (1988). Transcription of unrearranged antigen receptor genes in *scid* mice. EMBO J. **7:** 2019–2024.

Storb U. (1988). Immunoglobulin gene analysis in transgenic mice. *In:* Immunoglobulin Genes, Honjo T., Alt F.W., and Rabbits T.H., Eds. (New York: Academic Press), pp. 305–326.

Tidmarsh G.F., Heimfeld S., Whitlock C.A., Weissman I.L., and Muller-Sieburg C.E. (1989). Identification of a novel bone marrow-derived B cell progenitor population that coexpresses B220 and Thy-1 and is highly enriched for Abelson Leukemia Virus targets. Mol. Cell. Biol. **9:** 2665–2671.

Tsang H., Pinkert C., Hagman J., Lostrum M., Brinster R.L., and Storb U. (1988). Cloning of a gamma 2b gene encoding anti *P. aeruginosa* H chains and its introduction into the germline of mice. J. Immunol. **141:** 308–314.

Weaver D., Costantini F., Imanishi-Kari T., and Baltimore D. (1985). A transgenic immunoglobulin mu gene prevents rearrangement of endogenous genes. Cell **42:** 117–127.

Whitlock C.A., Robertson D., and Witte O.N. (1984). Murine B cell lymphopoiesis in long term culture. J. Immunol. Meth. **67:** 353–369.

Whitlock C.A., and Witte O.N. (1982). Long-term culture of B lymphocytes and their precursors from murine bone marrow. Proc. Natl. Acad. Sci. USA **79:** 3608–3612.

Whitlock C.A., Ziegler S.F., Treiman L.J., Stafford J.I., and Witte O.N. (1983). Differentiation of cloned populations of immature B cells after transformation with Abelson murine leukemia virus. Cell **32:** 903–911.

Witte O.N. (1983). Molecular and cellular biology of Abelson virus transformation. Curr. Top. Microbiol. Immunol. **13:** 127–146.

Witte P.L., Burrows P.D., Kincade P.W., and Cooper M.D. (1987). Characterization of B lymphocyte lineage progenitor cells from mice with severe combined immune deficiency disease (SCID) made possible by long term culture. J. Immunol. **138:** 2698–2705.

Wu Q., Tidmarsh G.F., Welch P.A., Pierce J.H., Weissman I.L., and Cooper M.D. (1989). The early B lineage antigen BP-1 and the transformation-associated antigen 6C3 are on the same molecule. J. Immunol. **143:** 3303–3308.

Yancopoulos G.D., and Alt F.W. (1986). Regulation of the assembly and expression of variable region genes. Annu. Rev. Immunol. **4:** 339–368.

